# The Factors Associated With Continuous Positive Airway Pressure (CPAP) Failure in Late Preterm and Term Infants and Its Impact on In-Hospital Outcomes

**DOI:** 10.7759/cureus.63895

**Published:** 2024-07-05

**Authors:** Bethany L Hunt, Amy Parikh, Deepak Jain

**Affiliations:** 1 Pediatrics, Rutgers Robert Wood Johnson Medical School, New Brunswick, USA; 2 Pediatrics, Nationwide Children’s Hospital, Columbus, USA

**Keywords:** failure, length of stay, morbidities, non-invasive respiratory support, late preterm

## Abstract

Background and objective

Late preterm and term infants commonly require continuous positive airway pressure (CPAP) on admission. However, CPAP failure in this population has not been well studied. Hence, we conducted this study to determine the impact of CPAP failure and identify antenatal factors associated with it in late preterm and term infants.

Materials and methods

We carried out a single-center retrospective analysis of all inborn infants of ≥34 weeks gestational age (GA) from 2012 to 2019 who received CPAP on admission to the neonatal intensive care unit (NICU). CPAP failure was defined as follows: escalation in the mode of respiratory support, surfactant administration, increase in FiO_2_ >0.2 above the baseline, or absolute FiO_2 _>0.4 for ≥3h; within 12h of admission. In-hospital outcomes and perinatal factors were compared between CPAP-failure and success groups. Multivariate stepwise binary logistic regression analysis (LRA) was used to assess the association between antenatal factors and CPAP failure.

Results

Of the 272 infants included in the study, 38 (14%) failed CPAP. Infants in the failure group received a longer duration of respiratory support [median (IQR): 3.0 (5.6) vs. 0.5 (0.5)d; p<0.001], and length of stay [9 (9) vs. 4 (4)d; p<0.001]. On LRA, higher GA was associated with reduced odds of CPAP failure. Maternal hypertensive disorders, meconium-stained amniotic fluid, and group B Streptococcus (GBS)-positive status were associated with increased odds of CPAP failure.

Conclusions

In this cohort of late preterm and term infants, CPAP failure was associated with worse in-hospital outcomes. Lower GA, maternal hypertensive disorders, meconium-stained amniotic fluid, and GBS-positive status were associated with CPAP failure. These data, if replicated in further studies, may help develop individualized respiratory support strategies.

## Introduction

Late preterm and term infants constitute the majority of admissions to the neonatal intensive care unit (NICU), with respiratory failure being one of the most common indications [1-3]. Continuous positive airway pressure (CPAP) is frequently used as the initial mode of respiratory support for managing respiratory failure. While there is significant evidence supporting the indications, outcomes, and risks for failure of CPAP in extremely preterm infants, there is a scarcity of such data related to late preterm and term infants [4-8].

The applicability of the available data to late preterm and term infants may be limited due to differences in lung maturation stages, causes and severity of respiratory failure, and more benign clinical course [3,9]. While some recent studies have evaluated non-invasive respiratory support in this patient population, they have been mostly limited to identifying the risk factors for requiring respiratory support, prediction of failure based on early postnatal factors, or have included different non-invasive respiratory support strategies [10-12]. The data on antenatal factors associated with failure of initial CPAP in this population is lacking and can potentially help identify at-risk populations and improve the management of respiratory failure in these groups.

In light of this, we conducted a retrospective study evaluating outcomes and antenatal factors associated with CPAP failure in late preterm and term infants. The objectives of this study were as follows: (1) to identify antenatal factors associated with CPAP failure in late preterm and term infants and (2) to determine the association between CPAP failure and in-hospital outcomes in this population.

## Materials and methods

We conducted a single-center, retrospective analysis of a cohort of all infants of ≥34w gestation born at Robert Wood Johnson University Hospital between January 2012 and December 2019 and admitted to the NICU on CPAP within 12h after birth. Infants with a major congenital anomaly and those receiving invasive mechanical ventilation on admission were excluded. For the study, CPAP failure was defined as any of the following within 12h of initiation of CPAP: escalation in the mode of support, need for surfactant, increase in Fio_2_ ≥0.2 above baseline for ≥3 hours, or absolute Fio_2_ ≥0.4 for ≥ 3 hours. All decisions regarding respiratory support were made by the medical team according to their clinical judgment with no standardized protocols. This study was approved by the Rutgers Human Research Protection Program Institutional Review Board (Study ID: Pro 2021001864) with a waiver of consent as it involved a retrospective analysis of de-identified data.

In-hospital respiratory and other outcomes were compared between CPAP-failure and success groups. These included mortality; pneumothorax; duration of respiratory support and oxygen supplementation; time to achieve full oral feeds; exposure to antibiotics; exposure to prolonged antibiotics, defined as >48h; and length of stay. The following antenatal and perinatal factors were compared between the groups: gestational age (GA), gender, maternal age, maternal smoking status, maternal diabetes, maternal hypertensive disorders (comprising chronic hypertension, pregnancy-induced hypertension, preeclampsia, and/or eclampsia), group B Streptococcus (GBS)-positive status, clinical chorioamnionitis, magnesium sulfate (MgSO_4_) exposure before delivery, any antenatal steroid exposure before delivery, meconium-stained amniotic fluid, small for gestational age (SGA) defined as birth weight <10th percentile, and mode of delivery. Postnatal variables included Apgar score, need for positive pressure ventilation (PPV) during resuscitation, mode of CPAP delivery at admission, and Fio_2_ on admission. 

Perinatal factors and in-hospital outcomes were compared between groups using Chi-square analysis for categorical variables, independent t-test for continuous variables, or nonparametric Mann-Whitney U test as determined by the Shapiro-Wilk test. Multivariate binary logistic regression analysis (MLR) was used to model the association between CPAP failure and antenatal risk factors. Data analysis was performed using SPSS Statistics (IBM Corp., Armonk, NY). A p-value <0.05 was considered statistically significant for all analyses.

## Results

Of the 272 infants who met the study inclusion criteria, 38 (14%) failed CPAP. Regarding the individual components of failure criteria, 68% of infants required escalation of the mode of respiratory support, 45% received surfactant, 37% met the criteria of absolute FiO_2_ >0.4, and 8% had an increase in FiO_2_ greater than 0.2 above baseline.

Maternal, perinatal, and demographic characteristics

There was no significant difference between the groups in maternal age, incidence of smoking, or maternal diabetes. A higher proportion of mothers in the CPAP-failure group had hypertensive disorders (32% vs. 16%; p = 0.019) with no difference in exposure to MgSO_4_. Both groups did not differ in antenatal risk factors for infection as determined by the incidence of GBS-positive status, prolonged rupture of membrane, or clinical chorioamnionitis (Table 1).

**Table 1:** Maternal characteristics of CPAP-failure and success groups CPAP: continuous positive airway pressure; GBS: group B Streptococcus; SD: standard deviation

Variables	CPAP failure (n = 38)	CPAP success (n = 234)	P-value
Maternal age, years, mean ± SD	30 ± 6.4	31 ± 6.4	0.547
Maternal smoking, n (%)	8 (21)	44 (19)	0.877
Maternal diabetes, n (%)	5 (13)	57 (24)	0.269
Maternal hypertensive disorders, n (%)	12 (32)	37 (16)	0.019
Maternal GBS-positive status, n (%)	12 (32)	41 (18)	0.053
Rupture of membrane >18h, n (%)	5 (13)	24 (10)	0.319
Clinical chorioamnionitis, n (%)	2 (5.3)	20 (8.5)	0.226
Exposure to MgSO_4_,n (%)	8 (21)	28 (12)	0.27
Any antenatal steroids, n (%)	8 (21)	53 (23)	0.827

The infants in the CPAP-failure group had lower GA [median (IQR): 37.1 (4.1) vs. 37.5 (3.1)w; p = 0.043] with no difference in birth weight or incidence of SGA. Both groups were similar in terms of female gender (34% vs. 44%; p = 0.257) and mode of delivery. While there was a higher proportion of infants in the CPAP-failure group with meconium-stained amniotic fluid (21% vs. 13%; p = 0.086), five-min Apgar score <5 (7.9% vs. 2.1%, p = 0.051), and need for positive pressure during resuscitation (82% vs. 74%; p = 0.313), they did not reach statistical significance. Infants in the failure group had a higher admission FiO_2_ [median (IQR): 0.35 (0.16) vs. 0.23 (0.09); p<0.001], and a smaller proportion of infants used bubble device for CPAP (87% vs. 97%; p = 0.006) as compared to the success group (Table 2). 

**Table 2:** Birth and admission characteristics of CPAP-failure and success groups CPAP: continuous positive airway pressure; IQR: interquartile range; SD: standard deviation

Variables	CPAP failure (n = 38)	CPAP success (n = 234)	P-value
Gestational age, weeks, median (IQR)	37.1 (4.1)	37.5 (3.1)	0.043
Birth weight, g, mean ± SD	2909 ± 608	3065 ± 602	0.14
Small for gestational age (birth weight <10th centile), n (%)	3 (7.9)	15 (6.4)	0.733
Female gender, n (%)	13 (34)	103 (44)	0.257
Delivery by Cesarian section, n (%)	19 (50)	136 (58)	0.348
Meconium-stained amniotic fluid, n (%)	8 (21)	30 (13)	0.086
Five-min Apgar score <5, n (%)	3 (7.9)	5 (2.1)	0.051
Need for positive pressure during resuscitation, n (%)	31 (82)	173 (74)	0.313
Admitted from nursery, v	4 (11)	36 (15)	0.433
Admission FiO_2_,median (IQR)	0.35 (0.16)	0.23 (0.09)	<0.001
Bubble device for CPAP use, n (%)	33 (87)	226 (97)	0.006

**Figure 1:**
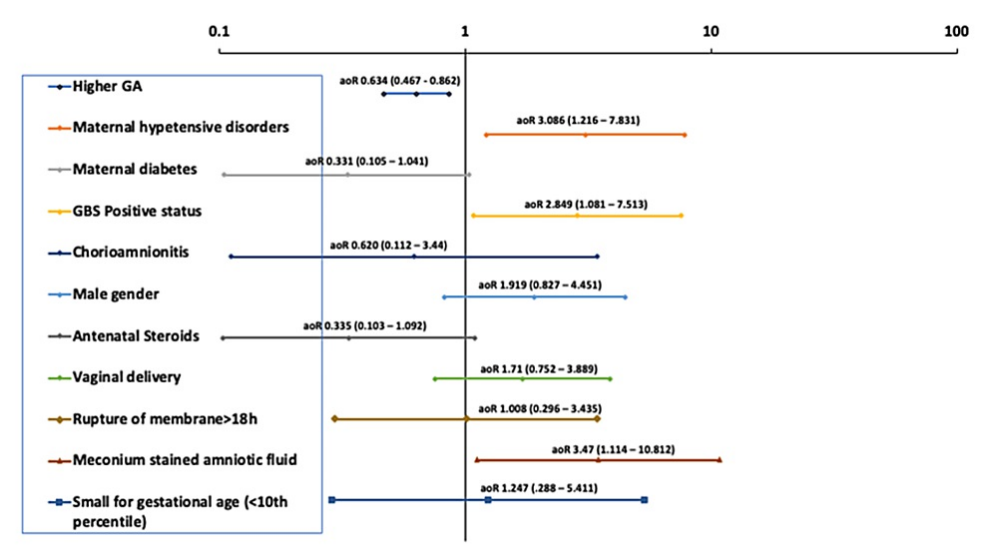
Multivariate logistic regression analysis of the association between initial non-invasive respiratory support failure and perinatal and demographic factors Adjusted odd ratios (aOR) with 95% confidence intervals (CI) are displayed for each factor GA: gestational age; GBS: group B Streptococcus

On MLR, higher GA was associated with lower odds of CPAP failure (aOR: 0.634, 95% CI: 0.467 - 0.862). As for antenatal factors, maternal hypertensive disorders (aOR: 3.086, 95% CI: 1.216 - 7.831), GBS-positive status (aOR: 2.849, 95% CI: 1.081 - 7.513), and meconium-stained amniotic fluid (aOR: 3.47, 95% CI: 1.114 - 10.812) were associated with higher odds of CPAP failure (Figure 1).

In-hospital outcomes 

No death occurred in either group. Infants in the CPAP-failure group had a longer duration of respiratory support [median (IQR): 3.0 (5.6) vs. 0.5 (0.5)d; p<0.001] and duration of oxygen exposure [median (IQR): 1.7 (5.0) vs. 0.5 (0.5)d; p<0.001]. A higher proportion of infants in the failure group received antibiotics (90% vs. 61%; p<0.001) and had a prolonged antibiotics course (37% vs. 9.7%; p<0.001). Finally, infants in the CPAP-failure group took longer to achieve full feeds [median (IQR): 6.0 (6.0) vs. 2.0 (3.0)d; p<0.001], and had a longer hospital stay than the success group [median (IQR): 9.0 (9.0) vs. 4.0 (4.0)d; p<0.001] (Table 3).

**Table 3:** In-hospital outcomes for CPAP-failure and success groups CPAP: continuous positive airway pressure; IQR: interquartile range

Outcome	CPAP failure (n = 38)	CPAP success (n = 234)	P-value
Duration of respiratory support, days, median (IQR)	3.0 (5.6)	0.5 (0.5)	<0.001
Duration of oxygen supplementation, days, median (IQR)	1.7 (5.0)	0.5 (0.5)	<0.001
Infants with pneumothorax, n (%)	7 (18)	22 (9.4)	0.188
Infants exposed to antibiotics, n (%)	34 (90)	142 (61)	<0.001
Infants on antibiotics >48h, n (%)	14 (37)	22 (9.4)	<0.001
Postnatal age at achieving full feeds, days, median (IQR)	6.0 (6.0)	2.0 (3.0)	<0.001
Length of hospital stay, days, median (IQR)	9.0 (9.0)	4.0 (4.0)	<0.001

## Discussion

In this cohort of late preterm and term infants receiving CPAP on admission to the NICU, 14% met the criteria for failure, with infants in the failure group requiring about six times the duration of respiratory support and twice the length of stay. While higher GA was associated with lower odds of CPAP failure, maternal hypertensive disorders, meconium-stained amniotic fluid, and GBS-positive status were associated with higher odds of CPAP failure. To our knowledge, this is the first study to examine antenatal risk factors and outcomes of CPAP failure in late preterm and term infants. Unlike some of the previous studies, we used 12h as the cut-off for defining CPAP failure, as, in our experience, most of these infants require escalation of respiratory support soon after birth. A potential critique of this study is the relatively arbitrary criteria for defining CPAP failure. These criteria were chosen to account for a lack of strict guidelines for the escalation of respiratory support and surfactant administration in this patient population. Using these criteria, the CPAP failure rate of 14% was similar to previously reported rates of 12% by Tournex et al. [10] and 11% by Son et al. [11].

Maternal hypertensive disorders were found to be associated with increased odds of failure of CPAP, with no difference in magnesium sulfate exposure or incidence of SGA in the current study. An association between preeclampsia and adverse neonatal respiratory outcomes has been previously described, with the biological rationale of anti-angiogenic in-utero environment in preeclampsia impairing alveolar and vascular development [13,14]. These data are more robust in very preterm infants [15-17] with limited and contradicting evidence in late preterm and term infants [18,19]. These contradictions could be attributed to variability in the anti-angiogenic profile of patients with preeclampsia at different points of gestation [20], variability in the severity of maternal hypertensive disorders, or the definition of respiratory morbidities in different studies. One of the limitations of the current study is that different maternal hypertensive disorders were combined in a single group, potentially diluting the overall impact on respiratory morbidities. Further larger studies are needed to better elucidate the role of individual types of maternal hypertensive disorders, degree of blood pressure control, or treatment strategies on neonatal respiratory morbidities.

In the current study, maternal GBS-positive status and meconium-stained amniotic fluid were associated with almost three times higher odds of CPAP failure. The association of CPAP failure with maternal GBS-positive status and meconium-stained amniotic fluid aligns with previous evidence on its association with neonatal morbidities [21,22]. The lack of association with chorioamnionitis, though interesting, is not entirely surprising as the impact of chorioamnionitis on neonatal morbidities has not been clearly defined [23,24]. As expected, CPAP failure in our study was associated with prolonged and more invasive respiratory support. The long-term impact of this small but significant increase in respiratory morbidities needs to be further evaluated in longer-term studies. More infants in the CPAP failure group were exposed to antibiotics and for a longer duration. Neonatal exposure to antibiotics has not only been linked to lower diversity in intestinal microbiome in the short term [25] but also to several longer-term morbidities like asthma [26], obesity [27], or autoimmune disorders [28]. Infants with CPAP failure on average were hospitalized for twice as long as the success group, and its impact on overall hospital costs and burden at the individual family level needs to be evaluated [29,30].

There are several limitations to the current study. The decisions related to changes in respiratory support were physician-dependent and could potentially be subjective. The single-center design of the study limits the generalizability of its findings. The retrospective nature and relatively small sample size of the study mean that all inferences from the study are hypothesis-building, which needs to be further tested in larger studies.

## Conclusions

Our single-center study showed that CPAP failure in late preterm and term infants was associated with adverse in-hospital respiratory outcomes, prolonged length of stay, and increased exposure to antibiotics. In addition to lower GA, maternal hypertensive disorders, meconium-stained amniotic fluid, and maternal GBS-positive status were associated with the failure of CPAP. These data, if replicated in further studies, may not only help clinicians in predicting the clinical course but also aid in developing individualized respiratory support strategies.

## References

[1] Mally PV, Hendricks-Muñoz KD, Bailey S (2013). Incidence and etiology of late preterm admissions to the neonatal intensive care unit and its associated respiratory morbidities when compared to term infants. Am J Perinatol.

[2] Sengupta S, Carrion V, Shelton J, Wynn RJ, Ryan RM, Singhal K, Lakshminrusimha S (2013). Adverse neonatal outcomes associated with early-term birth. JAMA Pediatr.

[3] Hibbard JU, Wilkins I, Sun L (2010). Respiratory morbidity in late preterm births. JAMA.

[4] Fischer HS, Bührer C (2013). Avoiding endotracheal ventilation to prevent bronchopulmonary dysplasia: a meta-analysis. Pediatrics.

[5] Stoll BJ, Hansen NI, Bell EF (2015). Trends in care practices, morbidity, and mortality of extremely preterm neonates, 1993-2012. JAMA.

[6] Flannery DD, O'Donnell E, Kornhauser M, Dysart K, Greenspan J, Aghai ZH (2016). Continuous positive airway pressure versus mechanical ventilation on the first day of life in very low-birth-weight infants. Am J Perinatol.

[7] Sweet DG, Carnielli V, Greisen G (2019). European Consensus Guidelines on the Management of Respiratory Distress Syndrome - 2019 update. Neonatology.

[8] Dargaville PA, Aiyappan A, De Paoli AG (2013). Continuous positive airway pressure failure in preterm infants: incidence, predictors and consequences. Neonatology.

[9] Escobar GJ, Clark RH, Greene JD (2006). Short-term outcomes of infants born at 35 and 36 weeks gestation: we need to ask more questions. Semin Perinatol.

[10] Tourneux P, Debillon T, Flamant C, Jarreau PH, Serraz B, Guellec I (2023). Early factors associated with continuous positive airway pressure failure in moderate and late preterm infants. Eur J Pediatr.

[11] Son H, Choi EK, Park KH, Shin JH, Choi BM (2020). Risk factors for BiPAP failure as an initial management approach in moderate to late preterm infants with respiratory distress. Clin Exp Pediatr.

[12] Suga S, Yasuhi I, Aoki M (2016). Risk factors associated with respiratory disorders in late preterm infants. J Matern Fetal Neonatal Med.

[13] Thébaud B, Lacaze-Masmonteil T (2010). If your placenta doesn't have it, chances are your lungs don't have it either: the "vascular hypothesis" of bronchopulmonary dysplasia starts in utero. J Pediatr.

[14] Parsons A, Netsanet A, Seedorf G, Abman SH, Taglauer ES (2022). Understanding the role of placental pathophysiology in the development of bronchopulmonary dysplasia. Am J Physiol Lung Cell Mol Physiol.

[15] Tagliaferro T, Jain D, Vanbuskirk S, Bancalari E, Claure N (2019). Maternal preeclampsia and respiratory outcomes in extremely premature infants. Pediatr Res.

[16] Rocha G, de Lima FF, Machado AP, Guimarães H (2018). Correction: preeclampsia predicts higher incidence of bronchopulmonary dysplasia. J Perinatol.

[17] Hansen AR, Barnés CM, Folkman J, McElrath TF (2010). Maternal preeclampsia predicts the development of bronchopulmonary dysplasia. J Pediatr.

[18] Tian T, Wang L, Ye R, Liu J, Ren A (2020). Maternal hypertension, preeclampsia, and risk of neonatal respiratory disorders in a large-prospective cohort study. Pregnancy Hypertens.

[19] Fratto VM, Ananth CV, Gyamfi-Bannerman C (2016). Late preterm neonatal morbidity in hypertensive versus normotensive women. Hypertens Pregnancy.

[20] Chaiworapongsa T, Romero R, Gotsch F (2023). Preeclampsia at term can be classified into 2 clusters with different clinical characteristics and outcomes based on angiogenic biomarkers in maternal blood. Am J Obstet Gynecol.

[21] Zhu Y, Huang J, Lin XZ, Chen C (2019). Group B Streptococcus colonization in late pregnancy and invasive infection in neonates in China: a population-based 3-year study. Neonatology.

[22] Hutton EK, Thorpe J (2014). Consequences of meconium stained amniotic fluid: what does the evidence tell us?. Early Hum Dev.

[23] Jobe AH (2012). Effects of chorioamnionitis on the fetal lung. Clin Perinatol.

[24] Nasef N, Shabaan AE, Schurr P, Iaboni D, Choudhury J, Church P, Dunn MS (2013). Effect of clinical and histological chorioamnionitis on the outcome of preterm infants. Am J Perinatol.

[25] Dardas M, Gill SR, Grier A, Pryhuber GS, Gill AL, Lee YH, Guillet R (2014). The impact of postnatal antibiotics on the preterm intestinal microbiome. Pediatr Res.

[26] Murk W, Risnes KR, Bracken MB (2011). Prenatal or early-life exposure to antibiotics and risk of childhood asthma: a systematic review. Pediatrics.

[27] Murphy R, Stewart AW, Braithwaite I, Beasley R, Hancox RJ, Mitchell EA (2014). Antibiotic treatment during infancy and increased body mass index in boys: an international cross-sectional study. Int J Obes (Lond).

[28] Cotten CM (2016). Adverse consequences of neonatal antibiotic exposure. Curr Opin Pediatr.

[29] Kirkby S, Greenspan JS, Kornhauser M, Schneiderman R (2007). Clinical outcomes and cost of the moderately preterm infant. Adv Neonatal Care.

[30] Rai P, Rani U (2019). Effect of newborn's admission to intensive care unit on "quality of life" of mother: an Indian perspective. J Matern Fetal Neonatal Med.

